# Recent Developments in Low-Level Lead Exposure and Intellectual Impairment in Children

**DOI:** 10.1289/ehp.6941

**Published:** 2004-04-28

**Authors:** Karin Koller, Terry Brown, Anne Spurgeon, Len Levy

**Affiliations:** ^1^Medical Research Council Institute for Environment and Health, University of Leicester, Leicester, United Kingdom; ^2^Institute of Occupational Health, University of Birmingham, Birmingham, United Kingdom

**Keywords:** children, cognitive function, intellectual impairment, IQ, lead exposure

## Abstract

In the last decade children’s blood lead levels have fallen significantly in a number of countries, and current mean levels in developed countries are in the region of 3 μg/dL. Despite this reduction, childhood lead poisoning continues to be a major public health problem for certain at-risk groups of children, and concerns remain over the effects of lead on intellectual development in infants and children. The evidence for lowered cognitive ability in children exposed to lead has come largely from prospective epidemiologic studies. The current World Health Organization/Centers for Disease Control and Prevention blood level of concern reflects this and stands at 10 μg/dL. However, a recent study on a cohort of children whose lifetime peak blood levels were consistently < 10 μg/dL has extended the association of blood lead and intellectual impairment to lower levels of lead exposure and suggests there is no safety margin at existing exposures. Because of the importance of this finding, we reviewed this study in detail along with other recent developments in the field of low-level lead exposure and children’s cognitive development. We conclude that these findings are important scientifically, and efforts should continue to reduce childhood exposure. However, from a public health perspective, exposure to lead should be seen within the many other risk factors impacting on normal childhood development, in particular the influence of the learning environment itself. Current lead exposure accounts for a very small amount of variance in cognitive ability (1–4%), whereas social and parenting factors account for 40% or more.

The effects of lead poisoning have been known since ancient times. In 200 BC the Greek physician Dioscorides observed that “lead makes the mind give way.” Until the beginning of the 20th century, lead poisoning was viewed largely as an occupational disease of adults. In the 1890s lead paint poisoning in children was first recognized, and childhood lead poisoning is now well documented and persists as a major public health problem throughout the world. Clinical features of acute lead poisoning include abdominal pain and neurologic symptoms of lead encephalopathy including headache and confusion. In severe cases renal failure and convulsions can occur (Lewis 1997), and extremely high levels may lead to coma and death (Meyer et al. 2003b). Features of chronic lead poisoning include behavioral changes, nephritis, and peripheral neuropathy [Lewis 1997; World Health Organization (WHO) 1995]. Children are more vulnerable to lead exposure for three reasons: young children are more at risk of ingesting environmental lead through normal mouthing behaviors (Lanphear et al. 2002), absorption from the gastrointestinal tract is higher in children than adults (Ziegler et al. 1978), and the developing nervous system is thought to be far more vulnerable to the toxic effects of lead than the mature brain (Lidsky and Schneider 2003).

Although there appears to be no dispute about the effects of high levels of lead, there has been uncertainty about the effects of low levels of lead exposure on children’s health. The debate has been particularly heated in the United States (Ferber 2002; Wakefield 2002), where data used to support laws and policies relating to lead exposure have become the subject of a number of lawsuits (Bellinger and Dietrich 2002; Mushak 2002; Needleman 2002; Nelson 2002; O’Dowd 2002; Pinder 2002). A special issue of *Archives of Clinical Neuropsychology* in 2001 was devoted to the topic of intelligence quotient (IQ) and low-level lead exposure in children. Five groups of scientists were invited to reply to an article by Kaufman (2001a) who posed the question “Do low levels of lead produce IQ loss in children?” (Brown 2001; Hebben 2001; Nation and Gleaves 2001; Needleman and Bellinger 2001; Wasserman and Factor-Litvak 2001). Kaufman argues that parental variables are far more important to a child’s cognitive development than is low-level lead exposure, and that the loss of a few IQ points (if true) is unlikely to have meaningful consequences for society (Kaufman 2001a, 2001b). In contrast, Needleman argues that lead-induced neurotoxicity has a causal role not only in cognitive loss but also in the subsequent development of juvenile delinquency and socially disruptive behavior (Needleman 1995; Needleman and Bellinger 2001; Needleman et al. 2002). These two positions represent the opposite ends of a spectrum of opinion on the relationship between low-level lead exposure and child development.

In contrast, debate in European countries has been muted with an overriding feeling that since the banning of leaded gasoline and lead-containing paints, lead exposure no longer poses a significant environmental threat to health. Publication of a study by Canfield and colleagues in 2003 (Canfield et al. 2003) challenged this view. Their study showed a dose-dependent decline in cognitive function in a cohort of children whose lifetime peak blood levels never rose above the current World Health Organization/Centers for Disease Control and Prevention (WHO/CDC) blood lead level of concern (10 μg/dL) and suggests there is no safety margin at existing exposures. Since its publication in April 2003, the Canfield study has been widely quoted and has extended the debate beyond the United States. With this in mind, the U.K. Department for Environment, Food and Rural Affairs commissioned the Medical Research Council Institute for Environment and Health to examine in detail the findings of Canfield and colleagues and to place their study within the context of other recent developments, not just in the area of low-level lead exposure but also in the wider context of normal childhood development. Our findings form the basis of this review.

## Sources of Lead Exposure/Current Blood Lead Levels

The main sources of lead in children’s environments are diet, lead-based paint in older housing, lead in soil and dust from contaminated leaded paint and gasoline, or past and present mining and industrial activity (Mielke 2002; Mielke and Reagan 1998). Exposure from air and waterborne sources has been greatly reduced with the introduction of unleaded gasoline and the replacement of lead water pipes and water tanks with nonlead alternatives. However, lead in soil and dust continues to be a major source of exposure. Indoor floor dust accounts for approximately 50% of a young child’s total lead intake [Institute for Environment and Health (IEH) 1998]. Although dust is a major source of lead intake throughout the first 1–2 years of childhood, lead-contaminated window sills in older housing become an increasingly important source of lead as children become mobile and stand upright.

Blood lead levels peak in children at around 2 years of age, and hand-to-mouth behavior and pica (eating substances not normally eaten e.g., soil or paint chips) are significantly associated with elevated blood lead levels (Lanphear et al. 2002). Children typically ingest < 50 mg/day of soil on average (Stanek and Calabrese 1995). However, in the case of pica, this amount can be ≥ 5 g a day (Mielke and Reagan 1998), and some children have ingested 25–60 g during a single day (Calabrese et al. 1997). Indeed, from the point of view of risk assessment, Calabrese and colleagues urge that soil pica be seen “as an expected, although highly variable, activity in a normal population of young children, rather than an unusual activity in a small subset of the population.” Soil abatement and paint hazard remediation programs have attempted to reduce children’s exposures to lead and other heavy metals, with mixed outcomes (Elias and Gulson 2003; Lanphear et al. 2003).

Children’s blood lead concentrations have fallen substantially in a number of countries in the last few decades, including the United States, Australia, Mexico, Germany, Poland, Sweden, and the United Kingdom (Delves et al. 1996; IEH 1998; Jarosinska and Rogan 2003; Meyer et al. 2003a). By 1999 the geometric mean blood lead for U.S. children 1–5 years of age had fallen from 15 μg/dL in the late 1970s to 2.0 μg/dL. A survey of 774 Swedish children over the period 1995–2001 showed blood lead levels had stabilized at 2 μg/dL at 7–11 years of age (Strömberg et al. 2003). In the United Kingdom, blood lead levels of 584 children measured during 1995 in the Avon Longitudinal Study of Pregnancy and Childhood (ALSPAC) showed a geometric mean of 3.44 μg/dL at 2.5 years of age (Golding et al. 1998). Despite these falls in blood lead levels, childhood lead poisoning continues to be a major public health problem for certain groups of children, specifically low-income, urban, African-American children in the United States (Roberts et al. 2001), children suffering from abuse and neglect (Chung et al. 2001), children living in rural mining communities (Lynch et al. 2000), and children in developing countries (Falk 2003; Fewtrell et al. 2004).

Lowering of exposure guideline levels reflects concern over the growing body of evidence that low levels of lead exposure have subtle effects on the nervous system of children. Since 1971 there have been four reductions in the CDC guideline level above which children are considered to have an elevated lead level. This level currently stands at 10 μg/dL (0.483 μmol/L). In 1997 the CDC estimated that 4.4% of children in the United States 1–5 years of age have blood lead levels ≥ 10 μg/dL (Lynch et al. 2000). In a recent report of blood lead levels in children 6 months to 5 years of age living in New Orleans, Louisiana, USA, 29% had levels ≥ 10 μg/dL (Rabito et al. 2003). In Wuxi City, China, 27% of children 1–5 years of age had blood lead levels > 10 μg/dL (Gao et al. 2001), whereas in Johannesburg, South Africa, the blood lead levels of 78% of schoolchildren ≥ 10 μg/dL (Mathee et al. 2002) and in Dhaka, Bangladesh, 87% of children 4–12 years of age had blood lead levels > 10 μg/dL (Kaiser et al. 2001). In the United Kingdom, large-scale blood lead monitoring programs ceased in the late 1980s, and there is a paucity of recent data on blood lead levels in young children. The proportion of children with blood lead levels > 10 μg/dL ranged from 0.74 to 5% according to recent reports from three different regions of England (IEH 1998; Lewendon et al. 2001; O’Donohoe et al. 1998), and there is growing concern that significant numbers of children under 5 years of age remain at risk from lead exposure in the United Kingdom (Grigg 2004).

## Cross-Sectional Studies

Cross-sectional studies form part of a worldwide effort to quantify the effects of lead exposure in children. The main limitation of such cross-sectional studies is that they measure blood lead at one specific time point only. Because the half-life of lead in blood approximates that of the erythrocyte (approximately 35 days), it is primarily an indicator of recent exposure. This is of particular importance with lead exposure, as blood lead levels peak in children at around 2 years of age.

We identified eight recent cross-sectional studies looking at the relationship between blood lead concentrations and children’s cognitive abilities: the large U.S. National Health and Nutrition Examination Survey (NHANES) III (Lanphear et al. 2000) and seven studies from six different countries [Croatia, Denmark, Saudi Arabia, Mexico, Pakistan, and Taiwan (Al-Saleh et al. 2001; Calderón et al. 2001; García-Vargas et al. 2002; Nielsen et al. 2000; Prpic-Majic et al. 2000; Rahman et al. 2002; Wang et al. 2002)]. All studies examined children 6 years of age or older (range 6–16 years) but differed in sample size (80–4,853) and the number of confounders considered. Mean blood lead levels ranged from 2.94 to 9.73 μg/dL. It was unfortunate that the very large NHANES III study (4,853 children) lacked data on two key confounders: home environment and maternal IQ. There was no consistent effect of blood lead levels on cognitive function across these studies, and taken together we believe they add little to the current debate on low lead exposure and its effect on cognition.

## Prospective (Longitudinal) Studies

The evidence for lowered intellectual and cognitive ability in children exposed to lead comes largely from prospective epidemiologic studies of cohorts in Boston, Massachusetts, USA; Cincinnati and Cleveland, Ohio, USA; Port Pirie and Sydney, Australia; and Yugoslavia (Factor-Litvak et al. 1999). A number of these studies are still ongoing. The main focus of current debate centers on the difficulties of adjusting for confounders (covariables), which include socioeconomic status (SES), home environment, and genetic factors. SES is measured in a number of ways that generally involve an index derived from data on household income, parents’ education, employment status, occupation, and home ownership. Home environment is frequently measured using the Home Observation for Measurement of the Environment Inventory (HOME) index. This reflects the quality and quantity of emotional and cognitive stimulation and support in the home environment. The total score is the sum of a number of items, each scored as present (1) or absent (0), in various categories: parental responsivity, acceptance of child, organization of home environment, provision of play materials, parental involvement with child, and variety of stimulation.

Longitudinal studies have many advantages over cross-sectional studies: *a*) the time sequence of events can be assessed, *b*) they can provide information on a wide range of outcomes, and *c*) there is reduced recall and selection bias compared with case–control studies. Children’s intellectual capacities change with time, and therefore age-specific tests must be used (i.e., there is no single psychometric test that can cover the entire age range of interest). Unfortunately, in the five ongoing lead/IQ studies identified, a variety of cognitive test instruments were used, even for children of the same age, and no two studies adjusted for the same covariables. It is therefore not possible to directly compare results between these studies.

The Yugoslavia Prospective Lead Study was initiated in 1985. Pregnant women (*n* = 1,502) living in two towns in Yugoslavia were identified as having differing lead exposures. One town is on the site of a lead smelter, whereas the other (control) town lies 25 miles to the south. Maternal blood lead was measured at midpregnancy and at delivery, and child blood leads were determined at subsequent 6-month intervals. The report by Wasserman and colleagues (2000) includes all children (*n* = 390) having at least one assessment of intellectual functioning at 3, 4, 5, or 7 years of age with complete data on all covariates. Three normed and age-specific tests of cognitive function were used. This review is a reanalysis of data given in the authors’ full 1999 report, when the study was in its fourteenth year (Factor-Litvak et al. 1999), and examines whether there are critical time periods for the effects of lead exposure on IQ. Data analysis was performed after grouping observations into three exposure–change categories. An increase in postnatal blood lead was defined by a change of 50% or more relative to prenatal levels, with the postnatal period divided into early (0–2 years of age) or late (2–7 years of age). Average prenatal blood lead levels were 10 μg/dL (range 3–30 μg/dL) and average postnatal levels at 2–7 years of age were 17.4 μg/dL (range 6.6–49 μg/dL). The wide ranges are a reflection of pooling data from children living in two towns with very different exposure levels.

Both prenatal (*p* < 0.001) and postnatal (*p* < 0.05) exposure were independently and significantly negatively correlated with IQ, and no critical period of vulnerability was found. A 50% rise in prenatal blood lead levels was associated with a 1.07-point loss in IQ score [95% confidence interval (CI), 0.6–1.53], whereas a 50% increase in postnatal blood lead relative to prenatal levels was associated with a 2.82-point IQ loss (95% CI, 0.52–4.91). Because the analyses first controlled for prenatal blood lead, the postnatal change measure indicated a substantial change in exposure and was not a reflection of whether the mean postnatal blood lead was high or low. Covariates included in the regression analysis were quality of the home (HOME score); maternal age, intelligence, education and ethnicity; birth weight; and sex. Together these accounted for approximately 50% of the variance in IQ at 7 years of age; lifetime lead exposure accounted for 4.2% of the variance (Factor-Litvak et al. 1999).

The report by Schnaas and colleagues (2000) forms part of the Mexico City Prospective Lead Study of 436 children born after uncomplicated pregnancy. Intellectual function was measured using the McCarthy Scale, which provides a general index of intellectual ability (General Cognitive Index, GCI); subtests measure both cognitive and motor function. Complete data were obtained for 112 children followed at 6-month intervals between 3 and 5 years of age. Prenatal blood lead measures were recorded at intervals during pregnancy, at delivery, and in cord blood. Average postnatal blood lead levels were calculated for three time periods: 6–18 months, 24–36 months, and 42–54 months. Geometric mean blood lead concentrations were approximately 10 μg/dL during the study period. Covariates used in regression models were maternal IQ, child’s sex, Apgar score at 5 min, birth weight, birth rank order, maternal educational level and IQ, and family SES (no details given). The authors did not include HOME scores, claiming that HOME scores are highly correlated with maternal IQ and it was therefore sufficient to include only maternal IQ in the model. The article is methodologically very complex, with interactions measured between many variables. The central finding is that prenatal log-transformed blood lead levels are not associated with intellectual function, either within or between subjects, whereas postnatal lead levels were significantly correlated with intellectual function. The strength of the association between mean blood lead (6–18 months) and GCI increases with age up to 4 years of age, after which it becomes less strong and decreases toward zero. This study is one of only a few that attempts to examine in detail the temporal pattern of the association of lead levels and intellectual function.

Tong et al. (2000) provide an update on the 375 children born in the lead-smelting city of Port Pirie, South Australia, who have been followed from birth and had reached 11–13 years of age at the time of the study. Previous studies of this cohort had shown that blood lead concentration was negatively associated with cognitive performance, with girls more sensitive to the effects of lead at 2, 4, and 7 years of age (Baghurst et al. 1992; Tong et al. 1996, 1998). Geometric mean blood lead levels in this cohort increased from 8.3 μg/dL at birth to 21.2 μg/dL at 2 years of age and decreased to 7.9 μg/dL at 11–13 years of age. This study explores whether there is any effect modification between lead exposure and key sociodemographic factors on IQ [measured using the Wechsler Intelligence Scales for Children-Revised (WISC-R) instrument] at 11–13 years of age. A large number of covariates were measured. Sociodemographic factors included sex, maternal IQ, HOME scores, and SES (estimated by Daniel’s scale of prestige of occupations in Australia). The cohort was divided into three groups on the basis of lifetime average blood lead levels, with the lowest group < 12 μg/dL and the highest group > 17 μg/dL. The effect of sex became statistically insignificant at 11–13 years of age, and the authors speculate that this may have been due to attrition in numbers. (The original cohort comprised 723 children.) The impact of lead on IQ was more marked in children with lower SES, although this became non-significant after adjusting for covariates. The high-SES children performed significantly better in arithmetic and vocabulary WISC-R subscales than children from poor SES backgrounds. Adjusted regression coefficients showed boys lost 2.6 IQ points (95% CI, 2.9 to –8.0), whereas girls lost 7.4 IQ points (95% CI, –1.7 to –13.1) for each 2.7-fold increase in lifetime average blood lead level.

The study by Emory and colleagues (2003) examined 79 mother–infant pairs who represented an independent sample drawn from a larger population of more than 500 subjects in an ongoing study of lead exposure. Mothers came from an urban cohort of low-SES African Americans in Atlanta, Georgia, USA, and their infants were included in the study if they were born after uneventful pregnancies. Maternal blood lead was measured at 6 months’ gestation and before delivery and compared with infant memory at 7 months, assessed by the Fagan preferential-looking test. This study was noteworthy for its use of more sensitive calibration standards and continual verification reference samples to increase confidence in measuring very low blood lead levels (< 5 μg/dL). Mean maternal blood lead was 0.72 ± 0.86 μg/dL. Umbilical blood lead was measured, but no data were given in the article. Infant Fagan scores were classified as low, medium, or high risk of later mental retardation. Significant negative correlations between maternal blood lead and subsequent infant Fagan ratings were reported. These differences were not related to gestational age, birth weight, or age at testing nor were they related to mother’s education, although it was not stated how this was measured. Overall, these findings should be treated cautiously because of the small numbers in the low- and high-risk groupings, and the lack of detailed information on confounders. The authors acknowledge that their results require replication. This is an ongoing study, and it will be of interest to follow future publications.

The recent article by Canfield and colleagues (2003) is part of this continuing evidence base and relates to a cohort of 240 children born between July 1994 and January 1995, living in Rochester, New York, USA, and enrolled in the Rochester Longitudinal Lead Study. This study population is a nested cohort within a larger group of children and their families who took part in a 24-month randomized dust-control trial published in 1999 (Lanphear et al. 1999). The article by Canfield and colleagues reports on the results of blood lead concentrations measured at 6, 12, 18, 24, 36, 48, and 60 months of age, and IQ determined at 3 and 5 years of age using the Stanford-Binet Intelligence Scale. The relations between blood lead levels and IQ were estimated with a variety of models, with adjustments for nine prespecified covariables: child’s sex, birth weight, iron status, and home environment (HOME scale conducted by face-to-face interview and direct observation within the home) (Canfield R, personal communication); mother’s IQ, years of education, race, and tobacco use during pregnancy; and household income. Adjusting for number of siblings and birth order did not alter the model estimates or significance levels, and these covariates were therefore not included in the secondary analyses (Canfield R, personal communication).

The study reports a significant negative association (*p* = 0.004) between blood lead levels and IQ, with a 0.46-point decrease in IQ for each microgram per deciliter increase in lifetime average blood lead concentration (lifetime being equivalent to the child’s total exposure over 3 or 5 years). For the subsample of children whose maximal blood lead level remained below 10 μg/dL over the 5 years, the IQ loss associated with a given change in blood lead level was greater. In these 101 children, the study indicates a loss of 0.74 IQ points for each microgram per deciliter increase in lifetime blood concentration. The authors suggest a nonlinear relationship between children’s IQ scores and their blood lead concentration, with larger associations at lower lead concentrations. The importance of this study is that it extends the association of blood lead concentrations and intellectual impairment to concentrations below the current level of concern, which stands at 10 μg/dL (0.483 μmol/L), and implies that there is no safety margin at existing exposures.

To fully evaluate the results of the Canfield study, the experiences of their (nested) study cohort within the original dust-control trial must be considered. The Canfield cohort comprised 240 children from a larger group of 276 children and their families taking part in the dust-control trial. Families were eligible for the dust-control trial if they lived in the city of Rochester and had a child 5–7 months of age at the time of the baseline visit. Participants were identified using sequential lists of live births from three urban hospitals, and families were recruited by telephone. Families who agreed to participate were visited by a study team who carried out a baseline interview and collected a venous blood sample from the child. In addition, an experienced technician collected and analyzed dust samples at various indoor locations and measured lead content of painted surfaces inside and outside the home. This original cohort was randomly divided into an intervention group (*n* = 140) and a control group (*n* = 135). Families in the intervention group received cleaning equipment and up to eight visits by a dust-control advisor, although the length of time between visits was not specified. All families continued to be visited by the study team at 6-month intervals for blood sampling and environmental lead measurements by a technician (blinded to intervention status). In addition, at each of these home visits an interviewer (also blinded) conducted a face-to-face interview to identify, among other things, the type and frequency of cleaning and the last time cleaning was performed.

Over the 18-month dust-control study period, there was a 2.6-fold increase in blood lead levels in all children, but no difference in blood lead levels by intervention status (geometric mean level 2.85 μg/dL at 6 months, 7.55 μg/dL at 24 months). House dust lead levels declined sharply in both the intervention and control groups. Six months after the first baseline visit, dust lead levels in interior window sills and on floors had decreased by approximately 50% and continued to decline at a slower rate over the following year. The authors recognized several limitations of the study, including sampling the same location in each house, that is, the act of sampling itself may have introduced an artificial decline in dust lead levels. Another possibility was that the act of sampling altered the cleaning behavior of the control group families (the Hawthorne effect). To examine whether the regular visits and dust sampling introduced such an effect, birth certificate data were used to construct a matched negative-control group of 236 children. Children were matched by race, month of birth, and poverty level (measured by census block group characteristics). At 24 months of age the geometric mean blood lead levels were 7.3 μg/dL (95% CI, 6.6–8.2) in the intervention group, 7.8 μg/dL (95% CI, 6.9–8.7) in the control group, and 7.3 μg/dL ± 2.2 μg/dL (CI not given) in the matched negative controls. No Hawthorne effect was apparent.

If it is assumed that the matched negative-control group lived in homes with dust lead levels equivalent to those found in the study cohort before any interventions (baseline values), house dust lead levels do not appear to correlate with blood lead levels in the study children. This is not discussed in the 1999 article by Lanphear et al. (1999) but highlights the difficulties of accurately measuring lead levels in the personal environment of young children. It is interesting that data on house dust lead levels were not included in a follow-up report on dust control and blood lead levels when these children attained 48 months of age (Lanphear et al. 2000).

Canfield et al. (2003) analyze the original intervention and the control children as a single population, and it is pertinent to ask whether any of the interventions in the original dust-control study may have affected blood lead or IQ levels in their (nested) study group. Although there was no significant difference in blood lead between the two groups from 12–24 months of age, the intervention group mean blood levels were 5–7% lower than the controls. If dust control had altered the variability in blood lead levels, this could have affected the power of the study to look at associations with IQ. The second question to ask is, “Could the up to eight extra visits from the dust-control advisor have resulted in a more stimulating learning environment for children in the intervention group compared with controls?” These families had extra visits by one of two randomly assigned advisors, with the provision and replenishment of cleaning equipment and supplies (brooms, dustpans, sponge mops, buckets, gloves, and detergents). Mean IQs of study children and their mothers were below the national average, commensurate with sample demographics. The IQs of the children were normally distributed, whereas IQs of the mothers were slightly skewed because of a larger than expected number of observations in the 70–75 range (Canfield R, personal communication). Because of low sample numbers no significance should be attached to this finding. If these interventions enhanced the cognitive development of the children, they would have resulted in a shift in the relationship between IQ and blood lead levels, with a higher IQ for a given lifetime blood lead level in half of the study children. The effect of this would be to reduce the overall contribution of lead exposure to intellectual impairment. Therefore, in the context of the results of the Canfield study, the previous experiences of their study cohort might have biased the study findings toward the null, that is, attenuated the association between blood lead level and IQ.

**Figure 1:**
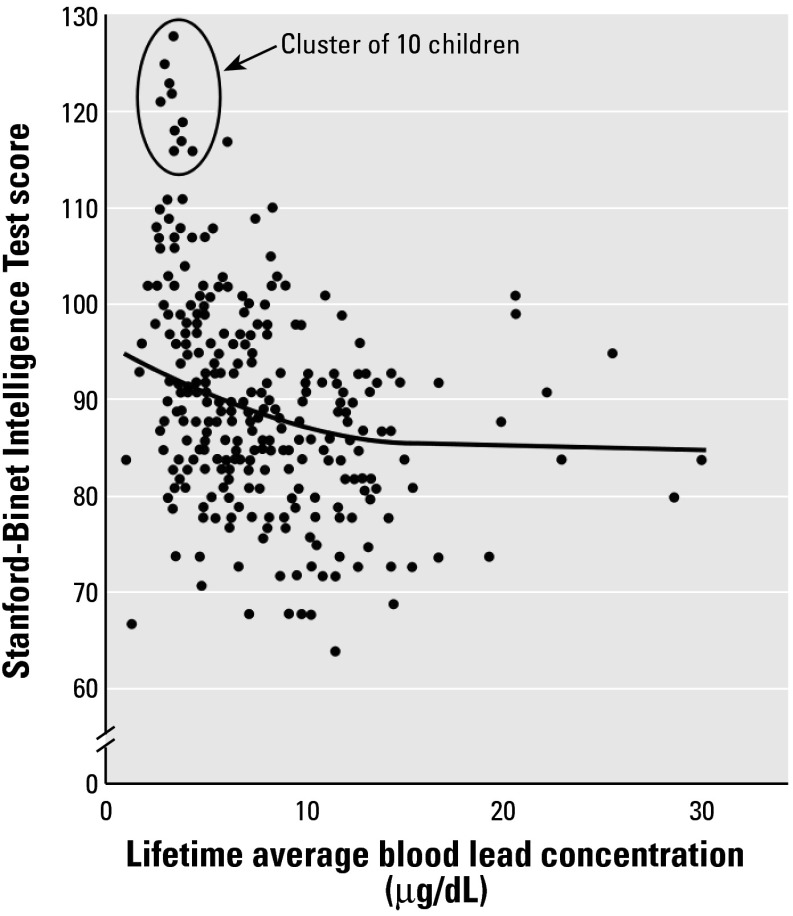
Intelligence quotient as a function of lifetime average blood lead concentration. Data were modified from Canfield et al. (2003).

Nonlinear mixed models were analyzed using the full range of blood lead values. Figure 1 illustrates the unadjusted lifetime average blood lead and IQ values. The authors state that the cluster of 10 children with low blood lead levels and high IQ “were not unduly influential in the statistical models,” and regression diagnostics did not identify any outliers in the data. Secondary analyses (using lifetime average blood lead levels) were carried out on the basis of observations with IQ scores < 110 (Canfield R, personal communication). For the full model this eliminated the 16 highest IQ scores. The overall linear regression coefficient for the remaining subgroup was –0.44 (*p* = 0.005), which is not significantly different from the coefficient of –0.46 for all 172 children. For the group of children with a peak blood lead concentration < 10 μg/dL, 15 observations were eliminated by using the IQ < 110 cutoff. In this case the linear regression coefficient was –1.07 (*p* = 0.038), which again is not significantly different from the coefficient of –1.37 for all children with a peak blood lead < 10 μg/dL. However, after eliminating these observations, the *p-*value decreased from 0.05 to 0.08 in the quadratic model.

It would be of interest to know *a*) if removal of data from the 140 children in the original dust-control intervention cohort alters the semiparametric analysis relationship given in Figure 1, and *b*) to which group the cluster of 10 children with high IQs (> 115) and low blood lead levels (< 5 μg/dL) were assigned in the original study. In addition, data from the nine children with the highest blood lead levels may have had a disproportionate influence on the final slope of the curve compared with subjects clustered around the average blood lead level, and further information on these nine children would also be of interest.

Cognitive function was assessed using an abbreviated Stanford-Binet Intelligence Scale (version IV) at 3 and 5 years of age, with a different examiner administering the test at each age. Results are expressed as the composite score. However, this test may not have been the most accurate measure of IQ for this cohort. The Stanford-Binet is heavily weighted on verbal skills and has been superseded by the Wechsler scales for this reason. Anyone who lacks English proficiency will do less well in this test, and children were correctly excluded from analysis if their parents lacked English proficiency. Overall, the study children had below-average Stanford-Binet scores (89.8 ± 11.4). However, the standard method for calculating the composite score excludes subtests with a raw score of zero, and thus overestimates IQ in those children achieving a zero in any subtest. The Stanford-Binet IV score at 3 years of age does not correlate well with Wechsler Preschool and Primary Scale of Intelligence (WPPSI) scores at 4–5 years of age, but correlation is significantly improved by considering the number of subtests the child did not perform at 3 years of age (Grunau et al. 2000). The power of this study would therefore have been increased if the children had been assessed using the WPPSI test or if the authors had considered the number of zero-scored subtests in their analysis. However, if it is assumed that children in the Canfield study achieving a zero in any subtest are those with below-average IQs, the overall effect would have been to introduce a differential error in the estimates of IQ, that is, overestimating the IQ scores of children with higher lead levels. This would have biased (toward the null) the estimate of the slope of the relationship between blood lead and IQ and would have reduced the nonlinearity observed in Figure 1.

In correspondence after publication of the Canfield study, Bellinger and Needleman (2003) reanalyzed data from their prospective Boston cohort study, focusing on 48 children whose blood lead levels never exceeded 10 μg/dL at birth, 6, 12, 18, 24, 57, or 120 months. The regression coefficient was greater (–1.56) than that derived from analyses of children with peak blood lead levels > 10 μg/dL (–0.58), that is, their results replicated those of Canfield and colleagues. This reproducibility is of particular interest because the Boston cohort (high SES, average IQ of 105 at 2–4 years of age, mean blood lead 6.5 μg/dL at 2 years of age) was in many respects very different from the Rochester cohort (low SES, average IQ of 90 at 3 years of age, mean blood lead 9.7 μg/dL at 2 years of age). The authors conclude that residual confounding probably accounts for at least some of the disparity between the regression coefficients above and below 10 μg/dL, and because of this “the precise shape of the dose–effect relation at lead levels below 10 μg/dL remains uncertain” (Bellinger and Needleman 2003).

In summary, of the three most recent longitudinal studies that measured prenatal lead exposure (average blood lead ranging from 1 to 11.5 μg/dL), two found a negative association with subsequent IQ, and one found no effect. In contrast, all five recent longitudinal studies that measured postnatal exposure (average blood lead levels ranging from 6 to 44 μg/dL) found significant associations with cognitive development, and this association was maintained after adjusting for a range of covariates including child’s sex and birth weight and parental/maternal IQ and years of education. The Port Pirie and Rochester studies considered the widest range of confounding factors and were the most robust methodologically. With the report of Canfield and colleagues and the recalculation of the Boston cohort results, these findings in nearly 1,300 children support an association between childhood lead exposure and subsequent cognitive impairment and extend the range of concern to children with lifetime average blood lead levels < 10 μg/dL.

## Discussion

Epidemiologic studies are subject to two types of error: systematic and random. Systematic errors (or bias) are by far the most problematic as they are generally not measured and they do not decrease as the sample size increases. Key sources of bias include those associated with aspects of selection and the distortion of the cause–effect relation by confounding. Reasons for the controversy over the lead–IQ link include *a*) the large number of confounders that must be considered when measuring an effect on children’s intelligence; and *b*) the frequent finding that the more covariates included in regression models, the smaller the effect of blood lead on IQ becomes, although it remains in the same direction (WHO 1995). The most important confounders are SES, parental IQ, and the quality of the home environment. Other factors associated with both IQ and blood lead levels include sex, nutritional status, and parental smoking behavior.

Three studies shed light on the area of confounding (Needleman and Bellinger 2001; Tong and Lu 2001;Wasserman and Factor-Litvak 2001). Blood lead levels have been negatively and positively associated with SES. Because of the sociodemographics and geography of Boston, Massachusetts, USA, increased prenatal lead levels were found in children of higher SES, and after adjustment for covariates the association of lead with IQ loss increased (Needleman and Bellinger 2001). This effect was also seen in the Yugoslavia prospective lead study in which children living near a smelter were from higher SES backgrounds than those living in a nearby control town with lower lead exposure (Wasserman and Factor-Litvak 2001). A study on the identification of confounders in the Port Pirie cohort study (Tong and Lu 2001) found that the size of the relationship between blood lead levels and mean IQ scores decreased by up to 40% when adjustment was made for 4 confounders but by less than 10% when a further 10 confounders were added to the regression models. The four main covariates were SES, quality of home environment, maternal IQ, and parental smoking behavior. The 10 extra confounders, which had little effect individually, included age, sex, birth weight, birth rank, maternal age, number of siblings, and duration of breastfeeding (Tong and Lu 2001).

Bellinger (2000) has argued that factors such as SES and sex should not be viewed solely as confounders but as effect modifiers as well. Unlike confounding, effect modification is a true characteristic of the association between an exposure and its end point. An example is the association between alcohol consumption and blood pressure, which varies in size depending on the modifying effects of the age, sex, and smoking status of the individual. Using data from the Boston prospective lead study, Bellinger showed that children from the lower half of the social class distribution demonstrated a decrease in performance at lower blood lead levels than children in the upper half of the distribution. However, this protective effect of higher SES was lost in the group of children with the highest cord blood lead levels. The author’s hypothesis is that at a given exposure level the magnitude of the estimated effect varies depending on the individual’s location on the social class continuum (Bellinger 2000). This idea is not new. In 1984 Winneke and Krämer (1984) showed the protective effects of higher SES on visual-motor performance deficits in lead-exposed children and concluded,

the common practice of merely removing the effects of confounding factors, such as SES, appears doubtful. . . . In addition, some of the inconsistencies in this area of research might be due to differential sampling of subgroups of lead-exposed children characterized by different levels of psychosocial adversity.

Intuitively, Bellinger’s hypothesis is very attractive and provides a possible explanation for the variability between ostensibly similar studies. In the lead field in the past, study results have been deemed right or wrong, usually on the basis of how the issue of confounding was handled. If dose–effect relationships are not independent of other host characteristics, it will be necessary to model three (or more)-way interactions. However, most prospective studies are designed with only enough statistical power to detect main effects and do not have the power to detect effect modification in subgroups of the main cohort. Bellinger urges a move away from broad, population-based cohorts toward a greater use of focused sampling frames, which should include adequate numbers within specific subgroups (Bellinger 2000). The report on the Port Pirie cohort at 11–13 years of age supports the hypothesis that children from socially disadvantaged backgrounds are apparently more sensitive to the effects of lead than children from higher SES families (Tong et al. 2000).

The powerful influence of SES on developmental outcome has been elegantly demonstrated in a report on school-age children born to mothers with heroin dependency (Ornoy et al. 2001). The study followed children born to mothers with heroin dependency raised at home or adopted at a very young age. These children were compared with groups of control children with average SES, children raised in families with a heroin-dependent father, or children born in families with low SES and high environmental deprivation. The children with environmental deprivation or raised at home by parents with heroin dependency had the lowest intellectual achievements. The adopted children had normal scores on the verbal WISC-R and on the Bender test, as well as normal reading and arithmetic skills, although they had a higher rate of attention deficit hyperactivity disorder than control children. Ornoy later extended this work to include two other high-risk cohorts: children born to mothers with diabetes and children born prematurely with low birth weights. Again he was able to demonstrate that a good home environment had a strong influence on subsequent intellectual abilities but not on motor skills or attention span (Ornoy 2003).

Animal models using spatial learning in rats have shown the protective effect of an enriched environment on lead-induced neurotoxicity (Schneider et al. 2001). Of particular relevance is a recent report on rats exposed to low levels of lead during early development, that is, from birth to weaning at day 21. This exposure produced a lasting deficit in spatial learning that could be completely reversed by raising the rats in an enriched environment after weaning. This reversal was accompanied by nerve growth factor gene induction and recovery of deficits in hippocampal glutamate receptor gene expression (Guilarte et al. 2003).

Genetic predisposition can also affect vulnerability to lead-induced neurotoxicity; this area of research has recently been reviewed by Lidsky and Schneider (2003). Three genes are currently believed to play a role in lead neurotoxicity: the *ALAD* gene, which codes for δ-aminolevulinic acid dehydratase; the vitamin D receptor (VDR) gene; and the hemochromatosis gene coding for a defective protein known as HFE. There are two forms of the ALAD protein, ALAD1 and ALAD2; lead has a higher affinity for ALAD2. Preliminary evidence has shown adolescents with the ALAD1 phenotype are more resistant to the effects of lead on behavior and attention than ALAD2 individuals. There are at least two alleles (*b* and *B*) and three variants of the VDR genotype, and among adults occupationally exposed to lead, *b* individuals have higher lead levels in blood and bone. Mutated HFE protein is known to cause hemochromatosis, in which large quantities of iron are deposited in internal organs. Because lead can be incorporated into processes requiring iron, polymorphisms in HFE might be expected to influence lead absorption. It is likely that future epidemiologic studies will include analysis of ALAD status and possibly other biomarkers.

Generally, no single epidemiologic study should be treated as the sole source of convincing evidence. The weight of evidence for any causal link comes when a number of studies using similar or preferably different methodologies in different populations reveal the same finding. In the low-level lead–IQ link, the balance has come down in favor of an association, with the methodologically sound study by Canfield et al. (2003) indicating that these effects are seen at peak blood lead levels below 10 μg/dL. Having established a valid association, the use of a number of the Bradford Hill criteria can assist in making causal inferences: temporal relationship (Does lead exposure precede the effect on cognition?), biological plausibility (Are there neurotoxic mechanisms to explain the effect of lead on cognition?), and biological gradient and strength (Is there a dose–response relationship, and if so, how strong is it?).

Evidence is increasing for a temporal relationship. The finding that 4–5 years of age is the critical period for manifestation of earlier (postnatal) lead exposure (Schnaas et al. 2000) might explain the wide variability in effects reported in cross-sectional studies that only looked at children 6 years of age or older. Further support for the critical period comes from the finding by Rogan et al. (2001) that chelation therapy in lead-poisoned children has no beneficial effect when given at 4–6 years of age.

Mechanistically, no unifying theory explains the neurotoxicity of lead or how lead might affect cognition. The ability of lead to substitute for calcium is a common factor underlying many of its toxic actions, including apoptosis and influences on neurotransmitter storage and release, second messengers, cerebrovascular endothelial cells, and glial cells. A variety of mechanisms may be important, and these are summarized in recent reviews by De Gennaro (2002) and Lidsky and Schneider (2003). Lead activates calmodulin, calcineurin, and protein kinase C at very low doses (Deng and Poretz 2002; Kern and Audesirk 2000). Glutamate receptors are thought to be involved in mediation of learning and memory, and changes in *N*-methyl-d-aspartate glutamate receptor subunits are observed in animals that show cognitive deficits induced by exposure to lead (Lau et al. 2002; Nihei and Guilarte 2001). Lead-induced decreases in hippocampal neurotrophic factor gene expression in rats can be reversed by raising the animals in an enriched environment (Schneider et al. 2001).

Concerning dose–response relationships, IQ tests are blunt measures of neurologic status, and blood lead is at best only a crude index of lead-induced neurotoxicity. However, a negative association has been found across groups of children from a range of populations around the world. Visual-motor tests and tests of attention are designed to assess more limited cognitive domains than IQ tests, and it is of interest that more consistent decreases have been reported for these measures in cross-sectional studies (Al-Saleh et al. 2001; Calderón et al. 2001; Walkowiak et al. 1998) and prospective studies (Dietrich et al. 1993; Tong et al. 1996; Wasserman et al. 1997). The bluntness of IQ tests to measure cognitive function is underlined by a study on five children who underwent left temporal lobectomy for epilepsy. Each patient experienced significant language-related cognitive loss after surgery, and these losses were clinically evident in four of the five patients. However, IQ testing alone did not reliably identify these deficits. Only one child showed a loss of verbal IQ; the other four children showed increases in verbal IQ (Dlugos et al. 1999).

It is clear that blood lead levels have fallen significantly over the last 40 years. During the 1970s, childhood blood lead concentrations of 40 μg/dL were not unusual. The available evidence suggests that mean blood lead levels are now in the range 2–4 μg/dL in the United States and much of Europe. Despite this reduction in lead exposure, it could be argued that current baseline blood lead levels continue to constitute a global public health risk, as preindustrial humans are estimated to have had 100- to 1,000-fold lower blood lead levels than the population of today (Owen and Flegal 1998). With the recent evidence demonstrating an inverse association between blood lead levels and cognitive function in children exposed to low levels of lead, there is no safety margin at existing exposures. Clearly, efforts must continue to minimize childhood exposure. However, we would urge that these efforts be seen in perspective. The magnitude of the lead–IQ dose–response relationship is small on a population basis and should be set against the far greater combined effect of SES status and quality of the caregiving environment. It has been argued that, instead of “chasing after an ever-receding lead threshold,” attention and funds should be focused on “the more complex social ills that are associated with continued lead exposure in a small segment of the population” (Gee and McKay 2002). Current lead exposure accounts for a very small amount of variance in cognitive ability (1–4%), whereas covariates such as social and parenting factors account for 40% or more (Weiss 2000).

## References

[b1-ehp0112-000987] Al-Saleh I, Nester M, DeVol E, Shinwari N, Munchari L, Al-Shahria S (2001). Relationships between blood lead concentrations, intelligence, and academic achievement of Saudi Arabian schoolgirls. Int J Hyg Environ Health.

[b2-ehp0112-000987] Baghurst PA, McMichael AJ, Wigg NR, Vimpani GV, Robertson EF, Roberts RJ (1992). Environmental exposure to lead and children’s intelligence at the age of seven years. The Port Pirie Cohort Study. N Engl J Med.

[b3-ehp0112-000987] Bellinger DC (2000). Effect modification in epidemiologic studies of low-level neurotoxicant exposures and health outcomes. Neurotoxicol Teratol.

[b4-ehp0112-000987] Bellinger DC, Dietrich KN (2002). Ethical challenges in conducting pediatric environmental health research [Introduction]. Neurotoxicol Teratol.

[b5-ehp0112-000987] Bellinger DC, Needleman HL (2003). Intellectual impairment and blood lead levels [Letter]. N Engl J Med.

[b6-ehp0112-000987] Brown RT (2001). Behavioral teratology/toxicology: how do we know what we know?. Arch Clin Neuropsychol.

[b7-ehp0112-000987] Calabrese EJ, Stanek EJ, James RC, Roberts SM (1997). Soil ingestion: a concern for acute toxicity in children. Environ Health Perspect.

[b8-ehp0112-000987] Calderón J, Navarro ME, Jimenez-Capdeville ME, Santos-Diaz MA, Golden A, Rodriguez-Leyva I (2001). Exposure to arsenic and lead and neuropsychological development in Mexican children. Environ Res.

[b9-ehp0112-000987] Canfield RL, Henderson CRJ, Cory-Slechta DA, Cox C, Jusko TA, Lanphear BP (2003). Intellectual impairment in children with blood lead concentrations below 10 μg per deciliter. N Engl J Med.

[b10-ehp0112-000987] Chung EK, Webb D, Clampet-Lundquist S, Campbell C (2001). A comparison of elevated blood lead levels among children living in foster care, their siblings, and the general population. Pediatrics.

[b11-ehp0112-000987] De Gennaro LD (2002). Lead and the developing nervous system. Growth Dev Aging.

[b12-ehp0112-000987] Delves HT, Diaper SJ, Oppert S, Prescott-Clarke P, Periam J, Dong W (1996). Blood lead concentrations in United Kingdom have fallen substantially since 1984. Br Med J.

[b13-ehp0112-000987] Deng W, Poretz RD (2002). Protein kinase C activation is required for the lead-induced inhibition of proliferation and differentiation of cultured obligodendroglial progenitor cells. Brain Res.

[b14-ehp0112-000987] Dietrich KN, Berger OG, Succop PA (1993). Lead exposure and the motor development status of urban six-year-old children in the Cincinnati Prospective Study. Pediatrics.

[b15-ehp0112-000987] Dlugos DJ, Moss EM, Duhaime AC, Brooks-Kayal AR (1999). Language-related cognitive declines after left temporal lobectomy in children. Pediatr Neurology.

[b16-ehp0112-000987] Elias RW, Gulson B (2003). Overview of lead remediation effectiveness. Sci Total Environ.

[b17-ehp0112-000987] Emory E, Ansari Z, Pattillo R, Archibold E, Chevalier J (2003). Maternal blood lead effects on infant intelligence at age 7 months. Am J Obstet Gynecol.

[b18-ehp0112-000987] Factor-Litvak P, Wasserman G, Kline JK, Graziano J (1999). The Yugoslavia Prospective Study of environmental lead exposure. Environ Health Perspect.

[b19-ehp0112-000987] Falk H (2003). International environmental health for the pediatrician: case study of lead poisoning. Pediatrics.

[b20-ehp0112-000987] Ferber D (2002). Toxicology. Overhaul of CDC panel revives lead safety debate. Science.

[b21-ehp0112-000987] Fewtrell LJ, Prüss-Ustün A, Landrigan P, Ayuso-Mateos JL (2004). Estimating the global burden of disease of mild mental retardation and cardiovascular diseases from environmental lead exposure. Environ Res.

[b22-ehp0112-000987] Gao W, Li Z, Kaufman RB, Jones RL, Wang Z, Chen XZ (2001). Blood lead levels among children aged 1 to 5 years in Wuxi City, China. Environ Res.

[b23-ehp0112-000987] García-Vargas GG, Rubio-Andrade M, Casillas E, Vera-Aguilar E, Cebrián ME (2002). Cognitive effects in lead exposed children from urban areas in the region Lagunera, Mexico [Abstract]. Toxicologist.

[b24-ehp0112-000987] Gee A, McKay C (2002). Childhood blood lead and cognition [Letter]. J Toxicol Clin Toxicol.

[b25-ehp0112-000987] Golding J Smith M Delves HT Taylor H 1998. The ALSPAC study on lead in children. In: Recent UK Blood Lead Surveys, Report R9 (Gompertz D, ed). Leicester, UK:MRC Institute for Environment and Health, 35–39.

[b26-ehp0112-000987] Grigg J (2004). Environment toxins; their impact on children’s health. Arch Dis Child.

[b27-ehp0112-000987] Grunau RE, Whitfield MF, Petrie J (2000). Predicting IQ of biologically “at risk” children from age 3 to school entry: sensitivity and specificity of the Stanford-Binet Intelligence Scale IV. J Dev Behav Pediatr.

[b28-ehp0112-000987] Guilarte TR, Toscano CD, McGlothan JL, Weaver SA (2003). Environmental enrichment reverses cognitive and molecular deficits induced by developmental lead exposure. Ann Neurol.

[b29-ehp0112-000987] Hebben N (2001). Low lead levels and neuropsychological assessment: let us not be misled [Commentary]. Arch Clin Neuropsychol.

[b30-ehp0112-000987] IEH 1998. Recent Blood Lead Surveys. Report R9. Leicester, UK:MRC Institute for Environment and Health.

[b31-ehp0112-000987] Jarosinska D, Rogan WJ (2003). Preventing lead poisoning in children: can the US experience inform other countries? The case of Poland. Cent Eur J Public Health.

[b32-ehp0112-000987] Kaiser R, Henderson AK, Daley WR, Naughton M, Khan MH, Rahman M (2001). Blood lead levels of primary school children in Dhaka, Bangladesh. Environ Health Perspect.

[b33-ehp0112-000987] Kaufman AS (2001a). Do low levels of lead produce IQ loss in children? A careful examination of the literature. Arch Clin Neuropsychol.

[b34-ehp0112-000987] Kaufman AS (2001b). How dangerous are low (not moderate or high) doses of lead for children’s intellectual development?. Arch Clin Neuropsychol.

[b35-ehp0112-000987] Kern M, Audesirk G (2000). Stimulatory and inhibitory effects of inorganic lead on calcineurin. Toxicology.

[b36-ehp0112-000987] Lanphear BP, Dietrich KN, Auinger P, Cox C (2000). Cognitive deficits associated with blood lead concentrations < 10 μg/dL in US children and adolescents. Public Health Rep.

[b37-ehp0112-000987] Lanphear BP, Hornung R, Ho M, Howard CR, Eberle S, Knauf K (2002). Environmental lead exposure during early childhood. J Pædiatr.

[b38-ehp0112-000987] Lanphear BP, Howard C, Eberly S, Auinger P, Kolassa J, Weitzman M (1999). Primary prevention of childhood lead exposure: a randomized trial of dust control. Pediatrics.

[b39-ehp0112-000987] Lanphear BP, Succop P, Roda S, Henningsen G (2003). The effect of soil abatement on blood lead levels in children living near a former smelting and milling operation. Public Health Rep.

[b40-ehp0112-000987] Lau WK, Yeung CW, Lui PW, Cheung LH, Poon NT, Yung KKL (2002). Different trends in modulation of NMDAR1 and NMDAR2B gene expression in cultured cortical and hippocampal neurons after lead exposure. Brain Res.

[b41-ehp0112-000987] Lewendon G, Kinra S, Nelder R, Cronin T (2001). Should children with developmental and behavioural problems be routinely screened for lead?. Arch Dis Child.

[b42-ehp0112-000987] Lewis RL 1997. Metals. In: Occupational and Environmental Medicine, 2nd ed. (LaDou J, ed). Stamford, CT:Appleton and Lange, 405–439.

[b43-ehp0112-000987] Lidsky TI, Schneider JS (2003). Lead neurotoxicity in children: basic mechanisms and clinical correlates. Brain.

[b44-ehp0112-000987] Lynch RA, Malcoe LH, Skaggs VJ, Kegler MC (2000). The relationship between residential lead exposures and elevated blood levels in a rural mining community. J Environ Health.

[b45-ehp0112-000987] Mathee A, von Schirnding YER, Levin J, Ismail A, Huntley R, Cantrell A (2002). A survey of blood lead levels among young Johannesburg school children. Environ Res.

[b46-ehp0112-000987] Meyer I, Hoelscher B, Frye C, Becker K, Wichmann HE, Heinrich J (2003a). Temporal changes in blood lead levels of children in east Germany. Int J Hyg Environ Health.

[b47-ehp0112-000987] Meyer PA, McGeehin MA, Falk H (2003b). A global approach to childhood lead poisoning prevention. Int J Hyg Environ Health.

[b48-ehp0112-000987] Mielke HW (2002). Research ethics in pediatric environmental health: lessons from lead [Commentary]. Neurotoxicol Teratol.

[b49-ehp0112-000987] Mielke HW, Reagan PL (1998). Soil is an important pathway of human lead exposure. Environ Health Perspect.

[b50-ehp0112-000987] Mushak P (2002). Studies of pervasive toxic contaminants in children: staying the ethical course [Commentary]. Neurotoxicol Teratol.

[b51-ehp0112-000987] Nation JR, Gleaves DH (2001). Low-level lead exposure and intelligence in children. Arch Clin Neuropsychol.

[b52-ehp0112-000987] Needleman HL (1995). Environmental lead and children’s intelligence. Studies included in the meta-analysis are not representative [Letter]. Br Med J.

[b53-ehp0112-000987] Needleman HL (2002). What is not found in the spreadsheets [Commentary]. Neurotoxicol Teratol.

[b54-ehp0112-000987] Needleman HL, Bellinger D (2001). Studies of lead exposure and the developing central nervous system: a reply to Kaufman. Arch Clin Neuropsychol.

[b55-ehp0112-000987] Needleman HL, McFarland C, Ness RB, Fienberg SE, Tobin MJ (2002). Bone lead levels in adjudicated delinquents. A case control study. Neurotoxicol Teratol.

[b56-ehp0112-000987] Nelson RM (2002). Appropriate risk exposure in environmental health research. The Kennedy-Krieger lead abatement study. Neurotoxicol Teratol.

[b57-ehp0112-000987] Nielsen U, Kamp JJ, Grandjean P, White RF (2000). Environmental lead exposure and neurodevelopmental outcome in Danish preschool children [Commentary]. Neurotoxicology.

[b58-ehp0112-000987] Nihei MK, Guilarte TR (2001). Molecular changes in glutamatergic synapses induced by Pb^2+^: association with deficits of LTP and spatial learning. Neurotoxicology.

[b59-ehp0112-000987] O’Donohoe J, Chalkley S, Richmond J, Barltrop D (1998). Blood lead in U.K. children—time for a lower action level?. Clin Sci.

[b60-ehp0112-000987] O’Dowd P (2002). Controversies regarding low blood lead level harm. Med Health R I.

[b61-ehp0112-000987] Ornoy A (2003). The impact of intrauterine exposure versus postnatal environment in neurodevelopmental toxicity: long-term neurobehavioral studies in children at risk for developmental disorders. Toxicol Lett.

[b62-ehp0112-000987] Ornoy A, Segal J, Bar-Hamburger R, Greenbaum C (2001). Developmental outcome of school-age children born to mothers with heroin dependency: importance of environmental factors. Dev Med Child Neurol.

[b63-ehp0112-000987] Owen BD, Flegal AR (1998). Blood lead concentrations in marine mammals validate estimates of 10^2^- to 10^3^-fold increase in human blood lead concentrations. Environ Res.

[b64-ehp0112-000987] Pinder L (2002). Commentary on the Kennedy Krieger Institute Lead Paint Repair and Maintenance Study. Neurotoxicol Teratol.

[b65-ehp0112-000987] Prpic-Majic D, Bobic J, Simic D, House DE, Otto DA, Jurasovic J (2000). Lead absorption and psychological function in Zagreb (Croatia) school children. Neurotoxicol Teratol.

[b66-ehp0112-000987] Rabito FA, Shorter C, White LE (2003). Lead levels among children who live in public housing. Epidemiology.

[b67-ehp0112-000987] Rahman A, Maqbool E, Zuberi HS (2002). Lead-associated deficits in stature, mental ability and behaviour in children in Karachi. Ann Trop Paediatr.

[b68-ehp0112-000987] Roberts JR, Reigart JR, Ebeling M, Hulsey TC (2001). Time required for blood lead levels to decline in nonchelated children. J Toxicol Clin Toxicol.

[b69-ehp0112-000987] Rogan WJ, Dietrich KN, Ware JH, Dockery DW, Salganik M, Radcliffe J (2001). The effect of chelation therapy with succimer on neuropsychological development in children exposed to lead. N Engl J Med.

[b70-ehp0112-000987] Schnaas L, Rothenberg SJ, Perroni E, Martinez S, Hernández C, Hernández RM (2000). Temporal pattern in the effect of postnatal blood lead level on intellectual development of young children. Neurotoxicol Teratol.

[b71-ehp0112-000987] Schneider JS, Lee MH, Anderson DW, Zuck L, Lidsky TI (2001). Enriched environment during development is protective against lead-induced neurotoxicity. Brain Res.

[b72-ehp0112-000987] Stanek EJ, Calabrese EJ (1995). Daily estimates of soil ingestion in children. Environ Health Perspect.

[b73-ehp0112-000987] Strömberg U, Lundh T, Schütz A, Skerfving S (2003). Yearly measurements of blood lead in Swedish children since 1978: an update focusing on the petrol lead free period 1995–2001. Occup Environ Med.

[b74-ehp0112-000987] Tong S, Baghurst P, McMichael A, Sawyer M, Mudge J (1996). Lifetime exposure to environmental lead and children’s intelligence at 11–13 years: the Port Pirie cohort study. Br Med J.

[b75-ehp0112-000987] Tong S, Baghurst PA, Sawyer MG, Burns J, McMichael AJ (1998). Declining blood levels and changes in cognitive function during childhood: the Port Pirie cohort study. JAMA.

[b76-ehp0112-000987] Tong S, Lu AY (2001). Identification of confounders in the assessment of the relationship between exposure and child development. Ann Epidemiol.

[b77-ehp0112-000987] Tong S, McMichael AJ, Baghurst PA (2000). Interactions between environmental lead exposure and sociodemographic factors on cognitive development. Arch Environ Health.

[b78-ehp0112-000987] Wakefield J (2002). The lead effect?. Environ Health Perspect.

[b79-ehp0112-000987] Walkowiak J, Altmann L, Krämer U, Sveinsson K, Turfeld M, Weishoff-Houben M (1998). Cognitive and sensorimotor functions in 6-year-old children in relation to lead and mercury levels: adjustment for intelligence and contrast sensitivity in computerized testing. Neurotoxicol Teratol.

[b80-ehp0112-000987] Wang CL, Chuang HY, Ho CK, Yang CH, Tsai JL, Wu TS (2002). Relationship between blood lead concentrations and learning achievement among primary school children in Taiwan. Environ Res.

[b81-ehp0112-000987] Wasserman GA, Factor-Litvak P (2001). Methodology, inference and causation: environmental lead exposure and childhood intelligence. Arch Clin Neuropsychol.

[b82-ehp0112-000987] Wasserman GA, Liu X, Lolacono NJ (1997). Lead exposure and intelligence in 7-year-old children: The Yugoslavia Prospective Study. Environ Health Perspect.

[b83-ehp0112-000987] Wasserman GA, Liu X, Popovac D, Factor-Litvak P, Kline J, Waternaux C (2000). The Yugoslavia Prospective Lead Study: contributions of prenatal and postnatal lead exposure to early intelligence. Neurotoxicol Teratol.

[b84-ehp0112-000987] Weiss B (2000). Vulnerability of children and the developing brain to neurotoxic hazards. Environ Health Perspect.

[b85-ehp0112-000987] WHO 1995. Inorganic Lead. International Programme on Chemical Safety, Environmental Health Criteria 165. Geneva:World Health Organization. Available: http://www.inchem.org/documents/ehc/ehc/ehc165.htm [accessed 30 March 2004].

[b86-ehp0112-000987] Winneke G, Krämer U (1984). Neuropsychological effects of lead in children: interactions with social backgriound variables. Neuropsychobiology.

[b87-ehp0112-000987] Ziegler EE, Edwards BB, Jensen RL (1978). Absorption and retention of lead by infants. Pediatr Res.

